# Formation of ag nanoparticles in CO_2_-responsive microemulsion fabricated by a bio-based surfactant based on rosin

**DOI:** 10.1038/s41598-025-28384-w

**Published:** 2025-12-29

**Authors:** Xinyan Yan, Ruiqin Deng, Wenxuan Zhang, Zhengchuang Zhao, Zheng Xing, Huimin Cheng, Shuang Luo, Xujuan Huang, Haifei Lu, Zhaosheng Cai

**Affiliations:** 1 School of Chemical and Chemistry, Yancheng Institute of Technology, Yancheng, 224051 Jiangsu Province China; 2 College of Urban Construction, Zhejiang Shuren University, Hangzhou, 310015 China

**Keywords:** Rosin, Microemulsion, CO_2_ responsive, Ag NPs, Recycle, Nanoparticles, Sustainability

## Abstract

This article reports a novel CO_2_ responsive W/O microemulsion stabilized by a rosin-derived surfactant MPANGG in combination with sodium dodecyl sulfate (SDS) at a mole ratio of 1:1 with n-butyl alcohol as co-surfactant. The average size of microemulsion was lower than 100 nm. This microemulsion had excellent CO_2_ responsive property. The system can be rapidly switched between emulsified and demulsified states by alternating CO₂/N₂ treatment.This unique property not only facilitates the synthesis of spherical Ag nanoparticles but also, more importantly, enables the complete recovery and direct reuse of the oil phase for multiple synthesis cycles. The formation of Ag NPs was confirmed by a characteristic UV–Vis peak at 420 nm, while TEM revealed the synthesized Ag nanoparticles have an average diameter of 11.7 ± 0.5 nm, confirming a high degree of size uniformity. Furthermore, following demulsification, the upper oil phase was successfully recycled and reused for subsequent nanoparticle synthesis, demonstrating the combined economic and environmental benefits of this methodology.

## Introduction

Nanoparticles with unique properties make them desirable in the electronic industry, biology, and materials science^1^. Among various nanoparticles, silver nanoparticles (Ag-NPs) have become the most popular one in recent decades^2,3^. Ag-NPs exhibit excellent antimicrobial activity even at a low concentration thanks to its large surface to volume ratio^4^. Moreover, they have shown immunological response and low cost^5^. Therefore, they play an important role in the biomedical application such as medical imaging, drug delivery, therapeutics, and molecular diagnostics^6^. Generally, the properties of Ag-NPs are dependent on their size, size distribution and shape^7^. In some applications, it is required that the Ag-NPs should have as small in diameter as possible^8^. Thus, the controlled preparation of the size, size distribution of Ag-NPs is a crucial task. In the last decades, many methods have been exploited for the formation of Ag-NPs such as biochemical reduction^9,10^, chemical reduction in inverse micelles^11^, radiation chemical reduction and chemical reduction of Ag-NPs in aqueous solution with stabilizing agents^12^. In these methods, as a soft technique, the microemulsion technique is one of the most important methods to prepare Ag-NPs^13^.

A microemulsion may be defined as a thermodynamically stable isotropic nanodomains of two immiscible liquids consisting of nano-size domains of one or both liquids in the other, stabilized by an interfacial film of surfactant^14^. The water is solubilized in the polar core, forming “water-pools”, whose radius *Rw* is dependent on the ratio of water to surfactant concentration *W*. Hence the Ag-NPs as synthesized in this “water-pools” have monodisperse size distribution and with smaller particle diameter. The size, size distribution and shape of the final Ag-NPs can be easily controlled^13,15^. However, microemulsion method reported previously only could produce a relatively low concentration Ag-NPs, while often require relatively high concentration of surfactant to reach sufficient interfacial coverage micro-emulsify levels of ingredients and to provide sufficient stability for practical storage requirements. As a result, it is expensive to produce Ag-NPs. Moreover, the breaking or destabilization of thermodynamically stable microemulsion present more difficult than that of thermodynamically unstable traditional emulsion, resulting that the large amounts of surfactant and organic solvent is difficult to remove and recover^16–19^. It is a challenge in research how recycle and reuse surfactant to reduce costs.

Recently, responsive emulsion stabilized by stimulus responsive surfactant, particles or both have attracted increasing attention. these responsive surface-active molecules can be switchable between being surface active and surface inactive under external stimuli for example pH^20–22^, CO_2_/N_2_^23,24^, temperature^25^, light^26^, redox^27^, and magnetic field^28^ etc. And then, the obtained emulsion stabilized by these surface-active molecules can be reversible between the stable and demulsification under external stimuli. For light responsive surfactants, their structures usually contain azobenzene or its derivatives. However, the azo compounds suffer from the difficulties in storage and are potential carcinogens^29^. For pH and redox responsive surfactants, the addition of extra chemical may cause polluted system concerns and may increase the process cost. For magnetic field responsive surfactants, the preparation process is complex and cost is high. So relatively speaking, among these stimuli, CO_2_ has been attracted more attention from researchers thanks to its biocompatible, nontoxic, inexpensive, and easily remove. Hatton^30^ reported a responsive microemulsion stabilized by ionic liquids. This ionic liquid-in-oil microemulsion reversibly switchable between clear and turbid states under exposure to and removal of CO_2_. Zhang^31^ reported a CO_2_ responsive microemulsion fabricated by introducing commercial N, N-dimethyl-N-dodecyl amine into a sodium dodecyl sulphate–butanol–heptane solution. This system can be switched reversibly between a stable microemulsion and the disruption upon alternate bubbling of CO_2_ and N_2_. Xu^32^ reported a CO_2_-responsive super-amphiphile (D-OA) assembled with Jeffamine D 230 and oleic acid (HOA) via electrostatic interaction while Xu^16^ developed a CO_2_ responsive O/W microemulsion, which can be rapid responses, by mixing this super-amphiphile with heptane, n-butyl alcohol, and water. However, in these reports, most of the surfactants are derived from fossil resource.

Due to the pressure from the dwindling fossil resources, exploiting surfactants from naturally occurring or their derivatives as building blocks has drawn attention widely. First, natural resources not only impart biocompatibility to surfactant, meeting the requirements for food and daily applications, but also many of them usually contain unique structures, which may endow the surfactants with specific performance. In the past decades, surfactants prepared from cellulose^33,34^, soap-nut oil^35^, humic acid^36^, starch^37^, vegetable oils^38,39^, glucose^40^, and so forth have been reported. Among the various renewable raw materials, pine rosin has a plethora of advantages thanks to its sustainable, low cast, abundance, easy chemical modifications especially its hydrophobic hydrogenated phenanthrene ring structure, and has been widely used in preparing surfactants^41–45^. Rosin is a kind of natural secreta of pine. The abietic acid, whose structure contains a latent conjugated double bond and a carboxylic acid group, is the main ingredient of rosin. Therefore, it can be used for further chemical modification easily^46,47^. What is more, it has been proved that the rigid structure of rosin showed the excellent aggregation ability. Therefore, rosin is a promising candidate for preparing biobased surfactant. Although there are many responsive rosin-based surfactants had been reported^48–50^, few reports have focused on microemulsions stabilized by these rosin-based surfactants, especially responsive microemulsions fabricated by a bio-based surfactant based on rosin.

Herein, a rosin-based CO_2_-responsive surfactant, maleopimaric acid glycidyl ester 3-dimethylaminopropylamine imide (MPANGG), has been synthesized and reported in our previous work^51^. A Novel CO_2_-responsive microemulsion are constructed by mixing MPANGG and SDS at a 1:1 molecular ratio. As an example of application, experiments on formation Ag-NPs are conducted at the room temperature using this CO_2_-responsive microemulsion. The obtained Ag-NPs by UV spectra and TEM are studied.

## Experimental section

### Materials

Rosin was purchased from Westech chemical co. LTD (China). N, N’-dimethyl propylene diamine and glycidol were purchased from Energy chemical. Absolute ethyl alcohol, dichloromethane and absolute methyl alcohol were purchased from Wuxi city Yasheng chemical co. LTD (China). Triethylamine was purchased from Nanjing chemical reagent co. LTD. (China). Maleic acid was purchased from Shanghai Lingfeng chemical reagent co. LTD. (China). The gas of CO_2_ and N_2_ were provided by Yancheng Guangyuan gas Co., LTD. 2 wt% AgNO_3_ aqueous solution, n-hexane and n-butyl alcohol were purchased from was purchased from Aldrich. They were all used as received.

### Synthesis of maleopimaric acid glycidyl ester 3-dimethylaminopropylamine Imide (MPANGG)

MPANGG was synthesized and purified followed our previous report^51^. Its detailed synthesis process was reported and its CO_2_ responsive behavior also had been proved in that work.

### Preparing of microemulsions

Both MPANGG and SDS serve as surfactants in this system. A mixed emulsifier (ME) stock solution was prepared by combining nonionic MPANGG with anionic SDS at a precise 1:1 molar ratio, ensuring an exact equivalence between the tertiary amine groups of MPANGG and the sulfonic acid groups of SDS. This stoichiometric balance is critical, as it enables the microemulsion to break down completely upon CO₂ introduction. The key mechanistic insight is that upon CO₂ exposure, MPANGG is protonated to form cationic MPANGGH⁺, which then combines with anionic SDS in a 1:1 ratio via noncovalent electrostatic interactions. This association results in the loss of surface activity for both components, effectively removing surfactants from the system and causing the microemulsion to break down.

Firstly, prepare a 0.5 mol·L^−1^ SDS solution. The density of 0.5 mol/L SDS is 1.018 g·cm^−3^ at room temperature. In SDS aqueous solution, m (H_2_O):m (SDS) was 6.06 g:1 g, therefore, the ME was m (H_2_O):m (SDS):m (MPANGG) = 6.06 g:1 g:2.345 g adding MPANGG equal to the molar amount of SDS.

The pseudoternary phase diagram was created by titrating co-surfactant n-butyl alcohol to a mixture of ME water solution and n-hexane at different mass ratios at the room temperature with the assistant of magnetic stirring. Microemulsions at different compositions was observed.

### CO_2_ responsive and size distribution of the W/O microemulsion

The reversible CO_2_ responsive of W/O microemulsion was accomplished by purging and removing of CO_2_ into and out of the system and their appearance photos were taken by digital camera. The drop size distributions of W/O microemulsion were analyzed using Zeta sizer Nano ZS (Malvern).

### Preparing and characterization of Ag-NPs

0.4 g ME, 0.6 g n-hexane, 1.1 g n-butyl alcohol and 0.13 g 2% AgNO_3_ aqueous solution were mixed to obtain a uniform, colorless and transparent microemulsion and kept away from light. At room temperature for 4 days away from light, 0.3 mL samples were diluted with 3.3 mL n-hexane at intervals during which the ultraviolet-visible (UV–Vis) spectra were measured while the same concentration of microemulsion was used as background. A drop of the microemulsions containing Ag-NPs was casted onto a Cu grid and the samples were dried under air prior to TEM imaging, and their transmission electron microscopy (TEM) pictures were performed using a Tecnai microscope.

## Results and discussion

### Pseudo ternary phase diagram

**Fig. 1:**
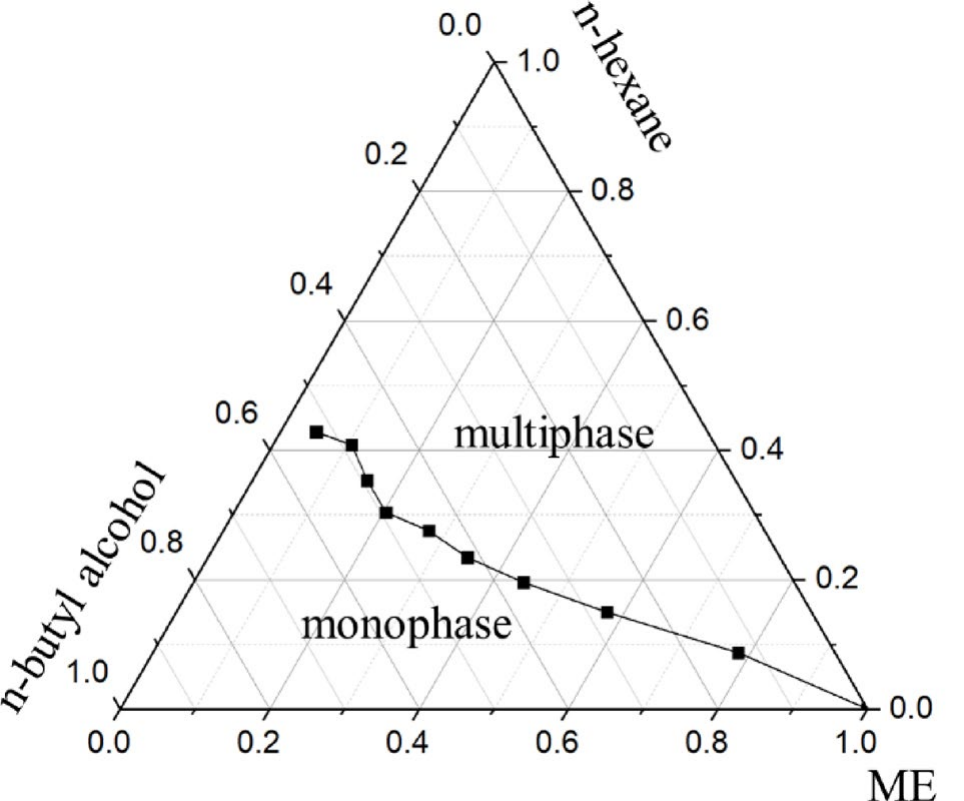
Pseudo-ternary phase diagram constructed at room temperature (25 °C) using the titration method with n-butyl alcohol as the co-surfactant.

The pseudo-ternary phase diagram was constructed at room temperature using the titration method. Briefly, mixtures with varying mass ratios of the aqueous phase (containing the mixed emulsifier, ME) and the oil phase (n-hexane) were prepared. Each mixture was titrated with the co-surfactant (n-butyl alcohol) until a uniform, transparent monophasic microemulsion formed instantaneously. The amount of n-butyl alcohol added was recorded. The resulting diagram is presented in Fig. 1. The region below the binodal curve represents the monophasic microemulsion zone, while the region above it corresponds to multiphase systems. The diagram clearly shows that the mixture composition dictates the extent of the single-phase and multiphase zones. Based on this diagram, a W/O microemulsion formulation comprising 0.4 g of ME and 0.6 g of n-hexane was selected for all subsequent investigations into its physicochemical properties and its application in the formation of Ag NPs.

### CO_2_ responsive behavior of W/O microemulsions

**Fig. 2:**
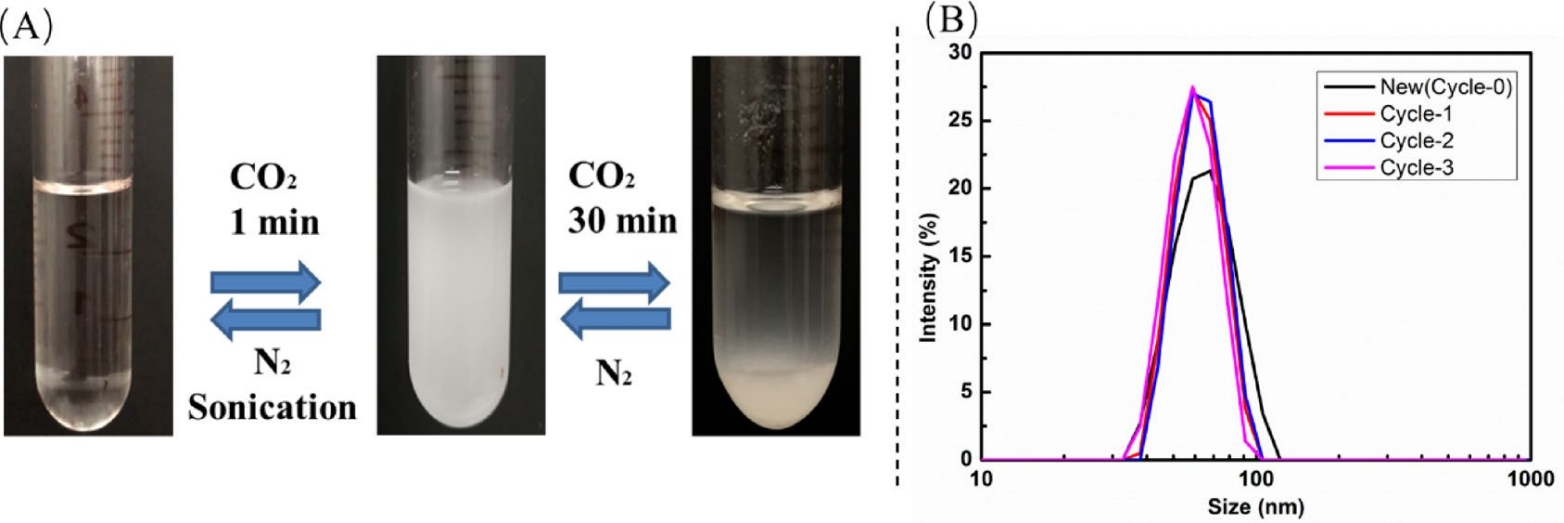
(**A**) The appearance (**B**) size distribution of water-in-oil microemulsion based on tertiary amines with CO_2_/N_2_ cycles.

In the absence of CO_2_, the W/O microemulsion behaves like a monophasic, homogeneous, transparent system (Fig. 2A left). To prevent the volatilization of n-butanol during bubbling gas, the operation was done at ice bath temperature. When CO_2_ was bubbled into the system, it turned opaque in less than 1 min (Fig. 2A middle), and then the homogeneous system separated into two phases upon bubbling CO_2_ for about 30 min (Fig. 2A right) meanwhile there was white solid precipitation appeared at the bottom bottle. Surprisingly, a single opaque phase was reversible changed back from two transparent phase of oil and water upon displacement of CO_2_ with N_2_ and then back to a transparent monophasic microemulsion under N_2_ continuously bubbled with assistant of sonication. The average size of microemulsion was lower than 100 nm indicating that this kind of emulsion belongs to the microemulsion level rather than traditional emulsion. Moreover, this microemulsion was formed spontaneously upon magnetic stirring or gentle shaking of the components without requiring high-energy input. Long-term stability studies showed no significant change in appearance over a period of 3 months at room temperature. Even more, it remained monophasic and clear without any phase separation even after high-speed centrifugation (15,000 rpm for 30 min) and through multiple freeze-thaw cycles. These results confirm that this system is a thermodynamically stable microemulsion. The unimodal distributions were observed from the particle distributions as shown in Fig. 2B implying the distribution of this microemulsion droplet was uniform. Moreover, its size distribution had little change after CO_2_/N_2_ response illustrating this microemulsion had excellent CO_2_ responsive property. In a word, the MPANGG-based W/O microemulsion could be reversibly destabilized and re-formed several times without any variation. The homogeneous and transparent W/O microemulsion could be still easily obtained even after several CO_2_/N_2_ cycles.

### Mechanism analysis of CO_2_ responsiveness of microemulsion

**Fig. 3:**
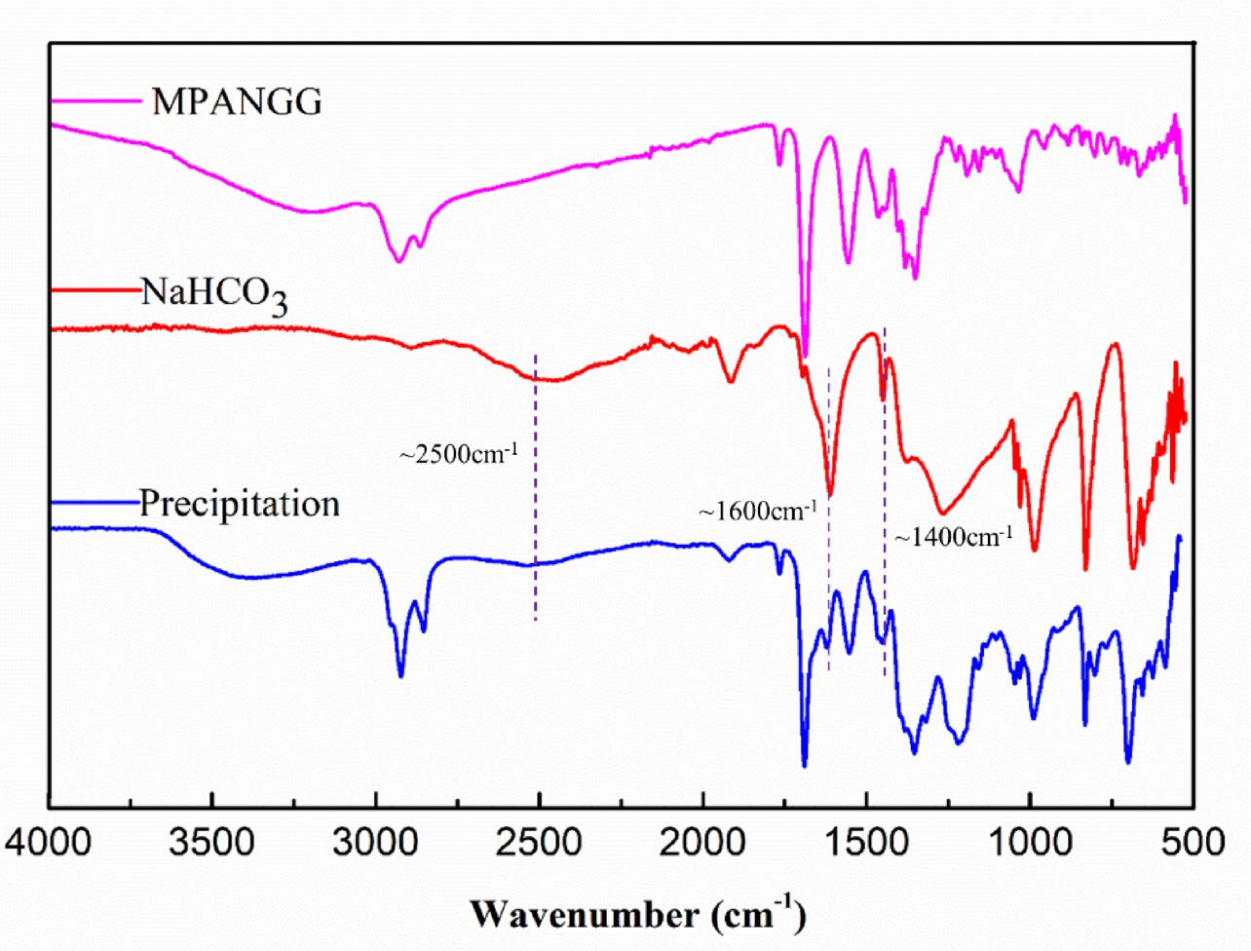
FT-IR spectra of the precipitation, NaHCO_3_ and MPANGG.

To explore the CO_2_ responsiveness mechanism of this microemulsion, the composition of the appeared white solid precipitation after bubbling CO_2_ into was analyzed. This white solid precipitation presents excellent water solubility, and a large number of bubbles were generated immediately as the drop of hydrochloric acid. Therefore, it was assumed that the precipitation maybe was hydro-carbonate or carbonate. As shown in Fig. 3, In the measured IR spectrum of NaHCO_2_, strong peaks at ~ 1600 cm^−1^ and ~ 1400 cm^−1^ would be observed clearly, while a broad peak in the 2500–2600 cm^−1^ region is usually the definitive evidence for confirming NaHCO_2_. The FT-IR spectra of the white solid precipitation were found to be consistent with that of the pure NaHCO_3_ exactly, except for the residual part of the surfactant MPANGG on its surface during the procedure of CO_2_ responsiveness. Therefore, it can be inferred that the white solid precipitation is NaHCO_3_ undoubtedly.

**Fig. 4:**
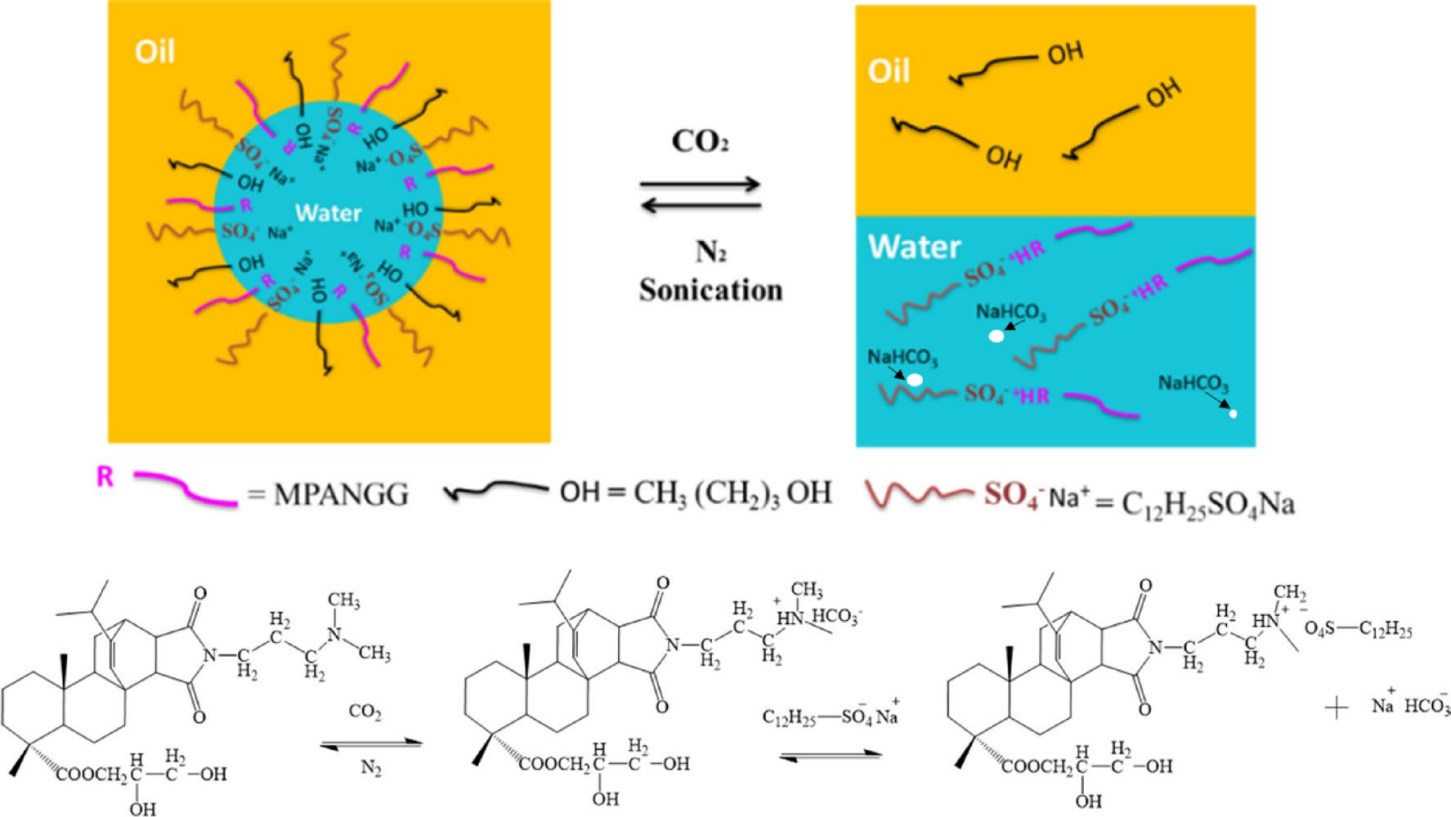
CO_2_/N_2_ response schematics of MPANGG-based W/O microemulsion.

Among the components of the microemulsion, MPANGG was CO_2_ responsive. During the introduction of CO_2_ into the MPANGG solution, the conductivity of solution increased to 104.5 µs/cm implying that the neutral tertiary amine group from MPANGG reacted with CO_2_ and H_2_O generating cationic tertiary amine bicarbonate surfactants (MPANGGH^+^)^51^. As the concentration of MPANGGH⁺ increases, the zeta potential rises, leading to electrostatic binding with anionic SDS. When N₂ is introduced to remove CO₂, the cationic MPANGGH⁺ is deprotonated and reverts to MPANGG. With increasing concentration of MPANGG, the zeta potential remains nearly zero, resulting in the loss of electrostatic interaction with SDS. The concentration-dependent zeta potential profiles of both MPANGG and MPANGGH⁺ was reported in our previous report. The reason for the formation of NaHCO_3_ was that cationic tertiary amine bicarbonate surfactants MPANGGH^+^ would combine with anionic surfactant SDS at a molecular ratio of 1:1 through noncovalent electrostatic interactions and small molecule NaHCO_3_, but the water content in the system was too low to dissolve all the generated NaHCO_3_, resulting in its supersaturation and precipitation. Procedure for the reaction was shown in Fig. 4. After MPANGGH^+^ and SDS combined, both of them lose their surface activity. There were no longer surfactants in the system, so the microemulsion broke down. After bubbling N_2_ to MPANGGH^+^ solution, the cationic tertiary amine carbonate MPANGGH^+^ has been converted back to the neutral tertiary amine-based surfactant MPANGG and the electrostatic interaction between MPANGGH^+^ and SDS disappeared. Therefore, with the assistant of sonication or magnetic stirring, vortex under N_2_ the supermolecule MPANGGH^+^/SDS would break back to small molecule MPANGG and SDS, which recovered their surface activity. And then, a transparent monophasic microemulsion was reversible changed back upon displacement of CO_2_ with N_2_ continuously bubbled with sonication. The conductivity cyclic change of MPANGG solution with CO_2_ bubbling into and out was also reported in our previous work^51^. This process could be repeated for several times without a significant change, which illustrated that the MPANGG had wonderful CO_2_ responsive behavior, resulting that this W/O microemulsion stabilized by MPANGG also present CO_2_ responsive behavior. The CO_2_/N_2_ response process diagram of -based also was shown in Fig. 4.

### Preparation of ag nanoparticles (Ag NPs) using this W/O microemulsion as the carrier

**Fig. 5:**
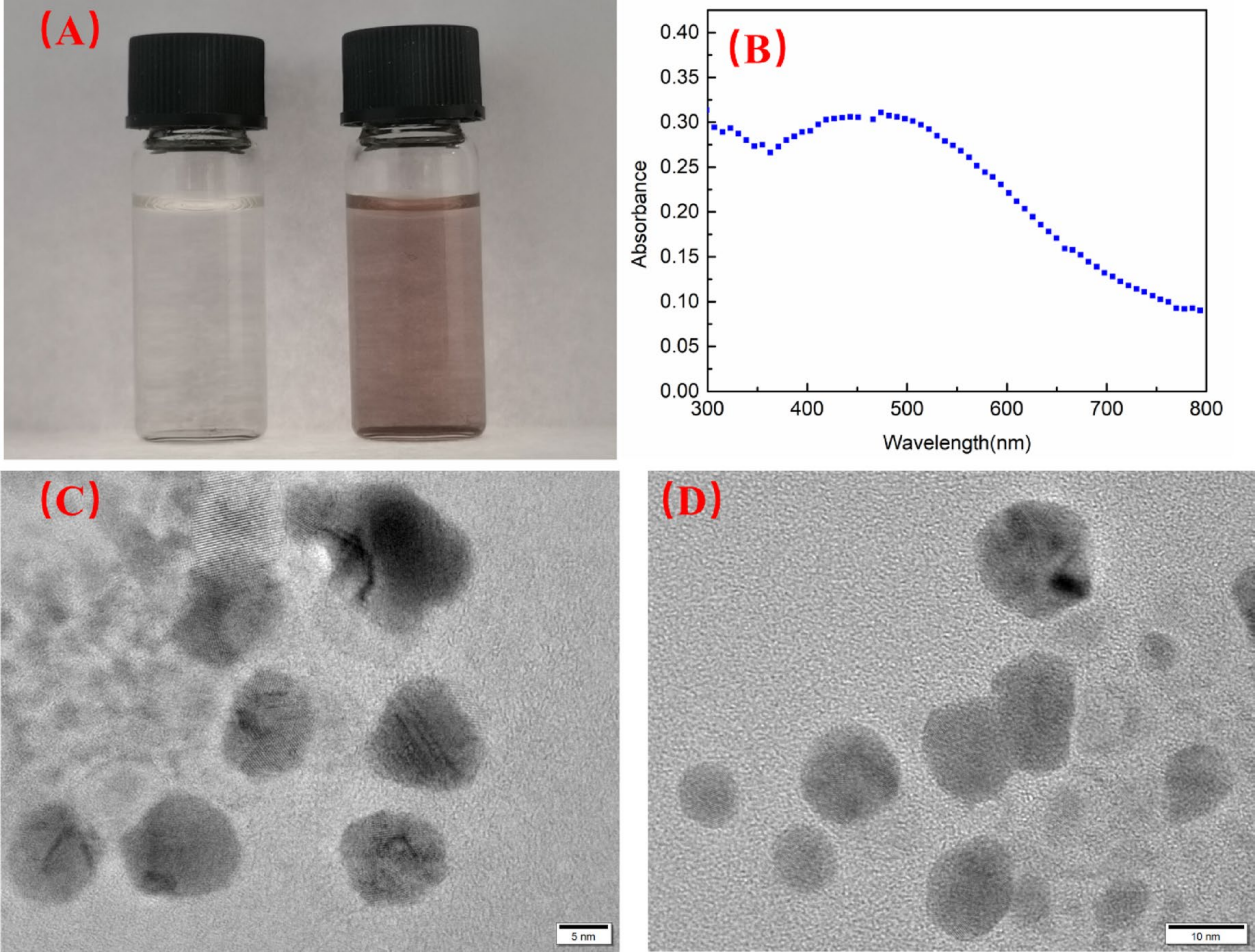
(**A**) Images of synthesis of Ag nanoparticles in microemulsion before reaction (left) and after reaction (right), (**B**) Uv–vis absorption spectra of Ag nanoparticles (**C**) TEM image of Ag nanoparticles formed in microemulsion. (**D**) TEM image of Ag nanoparticles formed in recycled microemulsion.

**Fig. 6:**
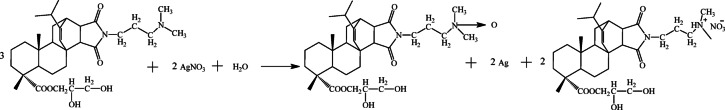
The possible schematic of reaction between tertiary amine from MPANGG and AgNO_3_.

Taking advantage of the nanostructures of W/O microemulsion, Ag nanoparticles (Ag NPs) were further prepared using this W/O microemulsion as the nanoreactor. Ag NPs are widely applied in microelectronics materials, catalysis, biomedicine and other fields thanks to their excellent photoelectric properties, catalytic properties and antibacterial properties. These properties mainly depend on particle size, particle size distribution and morphology of Ag NPs. Nanoparticles prepared by microemulsion method generally display the characteristics of small size and narrow particle size distribution. The microemulsion contains AgNO_3_ still is uniform, colorless and transparent as shown in Fig. 5A left, while gradually presents a change of red with the extension of the standing time of avoiding light but it remains uniform, colorless and transparent as shown in Fig. 5A right. The color change of the solution from colorless to light red implies that some Ag nanoparticles have been formed from the microemulsion containing AgNO_3_ aqueous solution because the AgNO_3_ is reduced to Ag NPs with small particle size by the tertiary amines in the system under the mild conditions at low temperatures. The possible schematic of reaction between tertiary amine from MPANGG and AgNO_3_ was shown in Fig. 6.

UV spectroscopy is a suitable method for following the nanoparticle formation as it gives information of both the kinetics and the morphology of the formed particles. The absorption of UV–Vis was caused by the plasma excitation of the surface electrons of Ag NPs due to the small size effect^52^. Therefore, the UV–vis spectrophotometer was used to detect the formation of Ag NPs further. As shown in Fig. 5B, UV–vis measurement results implied a high broad absorption band centered at approximately 420 nm, which is characteristic of Ag NPs^53^. The high broad absorption of UV–vis spectra mean fine nanoparticles yielded.

In addition, transmission electron microscopy (TEM) has further been applied to validate the existence of Ag NPs. As shown in Fig. 5C, there are a few fine spherical nanoparticles presented.

Statistical analysis of the TEM images reveals that the synthesized Ag nanoparticles have an average diameter of 11.7 ± 0.5 nm, confirming the Ag nanoparticles synthesized by this microemulsion present regular spherical shape and a high degree of size uniformity.

### CO_2_ responsive behavior of W/O microemulsions under the coexistence of ag NPs

The same as W/O microemulsion carrier, the microemulsion under the coexistence of Ag NPs also present excellent CO_2_ responsive behavior. The color change of the microemulsion contains AgNO_3_ from colorless to light red after four days avoiding light implying that the generation of Ag NPs. Injection of CO_2_ into Ag NPs microemulsion, the stable microemulsion was demulsified and then the homogeneous system separated into two phases meanwhile there was reddish-brown solid precipitation (mixture of NaHCO_3_ and Ag NPs) appeared at the bottom bottle. The solid precipitation was separated by centrifugation. After separation, the upper oil phase was collected and attempted to recovered and reused. Except water phase, the solid NaHCO_3_ produced by the reaction of MPANGGH^+^ with SDS also was discarded during the centrifugation separation process. Therefore, an equal amount of water and NaHCO_3_ must be replenished to the system and then N_2_ was bubbled to replace CO_2_. A single opaque phase was changed back and eventually the water and NaHCO_3_ disappear and become a uniform, clear, but light reddish-brown microemulsion again under N_2_ continuously bubbled with the assistant of sonication. 2 wt% AgNO_3_ was added into the regenerated microemulsion again, a new Ag NPs was reformed after storing 4 days avoiding light. The regenerated Ag NPs also were observed by TEM. As shown in Fig. 5D, there are a few fine spherical nanoparticles presented again with the average diameter of the resultant clusters is approximately 15 nm. The average diameters of Ag NPs both of the first generated and regenerated after CO_2_/N_2_ response cycle are all less than 20 nm. In other words, after microemulsion worked, the upper oil phase can be recycled and reused to prepare new Ag NPs again, so it not only has environmentally friendly properties, but also has economic benefits.

## Conclusion

In this work, a CO_2_/N_2_ responsive surfactant based on natural rosin maleopimaric acid glycidyl ester 3-dimethylaminopropylamine imide (MPANGG) was collaborated with sodium dodecyl sulfate (SDS) at a mole ratio of 1:1 as mixed emulsifier with n-butyl alcohol as co-surfactant to form a spontaneous stable water-in n-hexane microemulsion. Pseudo ternary phase diagram of [H_2_O/ SDS/ tertiary amine] - hexane -butanol system containing tertiary amine (MPANGG) were drawn, and the W/O microemulsion composition region was obtained. The MPANGG can be switched between cationic MPANGGH^+^ and nonionic item with bubbling CO_2_ / N_2_ to the solution. After bubbling CO_2_ into the MPANGG solution, neutral tertiary amine of MPANGG was protonated and formed cationic tertiary amine bicarbonate MPANGGH^+^. The cationic MPANGGH^+^ and anionic SDS at a mole ratio of 1:1 was assembled an assembling macromolecule via noncovalently electrostatic interactions, and then both of them lose surface activity leading to a phase separation of the microemulsion. Adversely, the cationic form MPANGGH^+^ was deprotonated and returned to the neutral form removing the CO_2_ from the system by bubbling N_2_ to the solution. The electrostatic interactions MPANGGH^+^ and SDS disappeared and the surface activity of MPANGG and SDS recovered. The phase-separated system was reversed into a transparent and spontaneous microemulsion. Therefore, this W/O microemulsions can be rapid switching responses upon the purge and removal CO_2_ of the system. This rapid switching behavior is helpful for the Ag nanoparticles preparation and separation. Ag nanoparticles with narrow particle size distribution were prepared using this W/O microemulsion as the carrier. Transmission electron microscopy (TEM) results show that the Ag nanoparticles present regular spherical shape and have a narrow size distribution. The microemulsion under the coexistence of Ag NPs also present excellent CO_2_ responsive behavior. After breaking emulsion, the upper oil phase can be recycled and reused to prepare new Ag NPs again, so it not only has environmentally friendly properties, but also has economic benefits. Besides, such microemulsion with rapid CO_2_-responsiceness and complete phase separation can have many potential applications such as contaminated soil cleaning, drill cuttings treatment and enhanced oil recovery.

## Data Availability

The datasets used and analyzed during the current study available from the corresponding author Haifei Lu ( [luhaifei@zjsru.edu.cn](mailto: luhaifei@zjsru.edu.cn) ) on reasonable request.
